# A conditional silencing suppression system for transient expression

**DOI:** 10.1038/s41598-018-27778-3

**Published:** 2018-06-21

**Authors:** Rodrigo Siqueira Reis, Celso G. Litholdo, Julia Bally, Thomas H. Roberts, Peter M. Waterhouse

**Affiliations:** 1 Department of Plant Molecular Biology, University of Lausanne, Lausanne, 1015 Switzerland; 2 School of Life and Environmental Sciences, University of Sydney, Sydney, NSW 2006 Australia; 3 Citrus Biotechnology Lab, Centro de Citricultura, Instituto Agronômico de Campinas, Cordeirópolis, SP 13490-000 Brazil; 4 Plant Breeding Institute, Sydney Institute of Agriculture, University of Sydney, Sydney, NSW 2006 Australia; 5 Centre for Tropical Crops and Biocommodities, Queensland University of Technology, Brisbane, QLD 4001 Australia

## Abstract

RNA silencing is a powerful tool deployed by plants against viral infection and abnormal gene expression. Plant viruses have evolved a suite of silencing suppressors for counter-defense, which are also widely used to boost transcript and protein accumulation in transient assays. However, only wild type silencing suppressor proteins have been reported to date. Here we demonstrate that P0 of Potato leafroll virus (PLRV), PLP0, can be split into two proteins that only show silencing suppression activity upon co-expression. We cloned each of these proteins in two different constructs and transiently co-infiltrated them in *N. benthamiana* leaves. We expressed a fluorescent protein from one of the vectors and observed that cells expressing both halves of PLP0 suppressed gene silencing. Further, we showed that Q system of *Neurospora crassa*, based on co-expression of a transcription activator and inhibitor, is functional in agroinfiltrated leaves of *N. benthamiana*. Q system combined with the split PLP0 system showed very tight co-expression of Q system’s transcriptional activator and inhibitor. Altogether, our experiments demonstrate a functioning conditional silencing suppressor system and its potential as a powerful tool for transient expression in *N. benthamiana* leaves, as well as the application of the Q system in plants.

## Introduction

Most plant viruses have RNA genomes and, as they replicate, long double-stranded RNAs (dsRNA) are produced and generically recognized by the host’s endonucleases, Dicer-like (DCL) enzymes. DCLs catalyze the formation of siRNAs that, upon loading into Argonaute (AGO) proteins, promote sequence-specific antiviral defense through RNA silencing^1^. This innate immune response is remarkably potent and versatile, and has driven most (if not all) plant viruses to evolve viral suppressors of RNA silencing (VSRs) as a counter-defense mechanism.

VSRs have also been widely exploited as a means to repress the host’s antagonistic effect on transgenic expression, particularly in transient assays. The Tomato bushy stunt virus (TBSV)-encoded P19 is the most commonly used VSR in transient assays. P19 suppresses RNA silencing, thereby aiding higher levels of transgenic-encoded proteins, by sequestering siRNAs in a manner that is neither sequence-specific nor organism-dependent^2^. This property of effectively preventing siRNAs loading into AGO proteins has made P19 an essential component in most transient assays, such as those using *Nicotiana benthamiana* leaves for Agrobacterium infiltration^3^. Potato leafroll virus (PLRV), a member of the luteovirus group and a major virus threat to potato crops, produces another well-characterized VSR protein, P0 (hereafter PLP0). PLP0 is an F-box protein that ubiquitinates Argonaute 1 (AGO1), the main RNA-silencing effector in plant defense against viruses, which is then selectively eliminated via autophagy^4,5^. Although less commonly used in transient assays than P19, PLP0 has been shown to be as effective as P19 as a silencing suppressor in transient assays using *N. benthamiana* leaves^6,7^.

Here we present a conditional silencing suppressor system based on split PLP0 proteins. We observe that PLP0 can be split into two functional proteins that are ineffective as silencing suppressors on their own, but upon co-expression show activity similar to that of PLP0 full-length protein. We applied this system to co-infiltration assays in *N. benthamiana* leaves by inserting each half of PLP0 into two separate vectors. We then expressed a fluorescent protein from one of the vectors and showed tight correlation of the fluorescence levels with the presence of both PLP0 halves in the cells. We further show that the binary system of *Neurospora crassa*, Q system, is functional in *N. benthamiana* agroinfiltrated leaves and, when combined with the conditional silencing suppressor PLP0 system, results in very tight co-expression of each of its binary components for transcriptional regulation. Hence, this work presents a conditional silencing suppressor system as a powerful tool for transient co-expression assays in *N. benthamiana* leaves, and demonstrates the feasibility of the Q system in plants.

## Results

### Split PLP0 retains silencing suppressor activity

**Figure 1:**
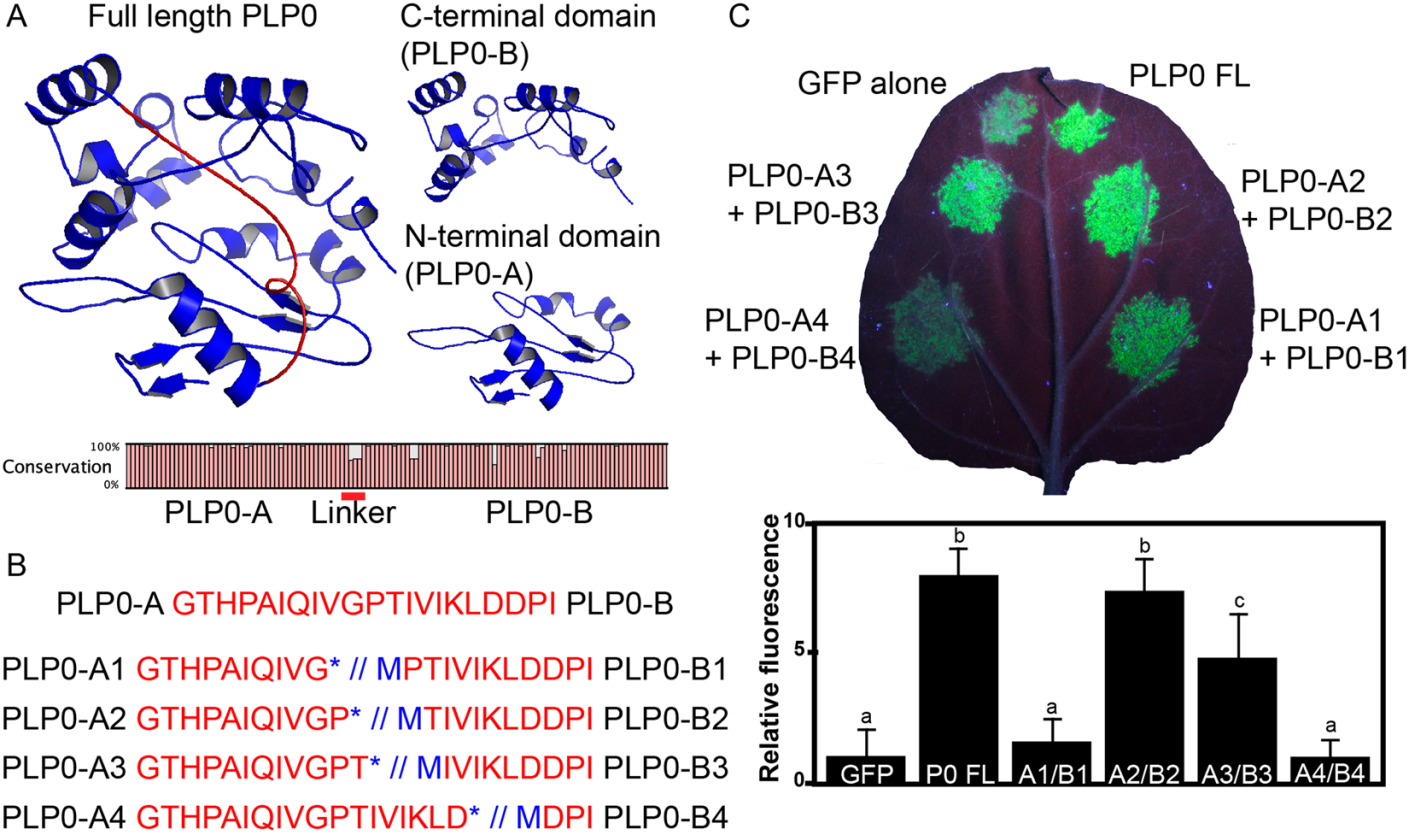
PLP0 putative domains and silencing suppression activity. (**A**) Predicted PLP0’s tertiary structure revealed two domains (blue), herein referred to as PLP0-A (N-terminal domain) and PLP0-B (C-terminal domain), separated by a linker (red). Amino acid conservation of PLP0 among PLRV isolates (bottom of panel) shows relatively low conservation of the predicted linker (red line). (**B**) Linker amino acid sequence (top of panel) and artificially introduced STOP (asterisk) and START (blue “M”) codons (bottom of panel) used to produce combinations of PLP0-A (PLP0 N-terminus) and PLP0-B (PLP0 C-terminus) proteins. (**C**) Representative *N. benthamiana* leaf co-infiltrated with GFP and PLP0 and each combination of PLP0-A and B (top of panel). Optical density (OD) was 0.2 per construct and analysed at 5 days post-infiltration (dpi). Fluorescence quantification of five biological replicates was performed using Fiji software and values are relative to GFP alone (bottom of panel). Pairwise comparisons (t-test; p-value < 0.05) are indicated by letters above bars; i.e., different letters indicate statistical differences in fluorescence levels.

PLP0 encodes an F-box protein that interacts with the host ubiquitination machinery and its target protein AGO1, promoting the degradation of this major silencing effector protein^4,8^. This raises the possibility that PLP0 may have at least two different domains or surfaces for protein-protein interactions with (i) the ubiquitination machinery and (ii) its target protein AGO1. Indeed, a predicted structure for PLP0 showed two putative domains, herein referred to as PLP0-A (amino acid residues 1–90) and PLP0-B (112–246), linked by an unstructured amino acid sequence (91–111) (Fig. 1A). Consistent with a previous report showing that the F-box domain of P0 from Cucurbit aphid-borne yellows virus is located within its N-terminus^5^, the PLP0-A sequence was found to be highly conserved among PLRV isolates (Fig. 1A and Table S1). However, the putative linker sequence showed a distinctly lower level of conservation, consistent with a role in joining two domains, and thus under lower evolutionary pressure. We then produced a series of constructs in which the putative linker was disrupted in various positions to produce two independent, but complementary, proteins (Fig. 1B). Each complementary combination was co-infiltrated with GFP in *N. benthamiana* leaves (Figs 1C and S1). Most combinations resulted in some level of silencing suppression, with the PLP0-A2 and PLP0-B2 co-infiltrated combination resulting in silencing suppression levels similar to that of full-length PLP0, based on the greatest increase in GFP accumulation relative to the control (without addition of silencing suppressor). Hence, these results support our predicted PLP0 folding structure consisting of two domains, and provide the means to split PLP0 into two functionally complementary proteins.

### Analysis of co-infiltration assays using the split PLP0 proteins

**Figure 2:**
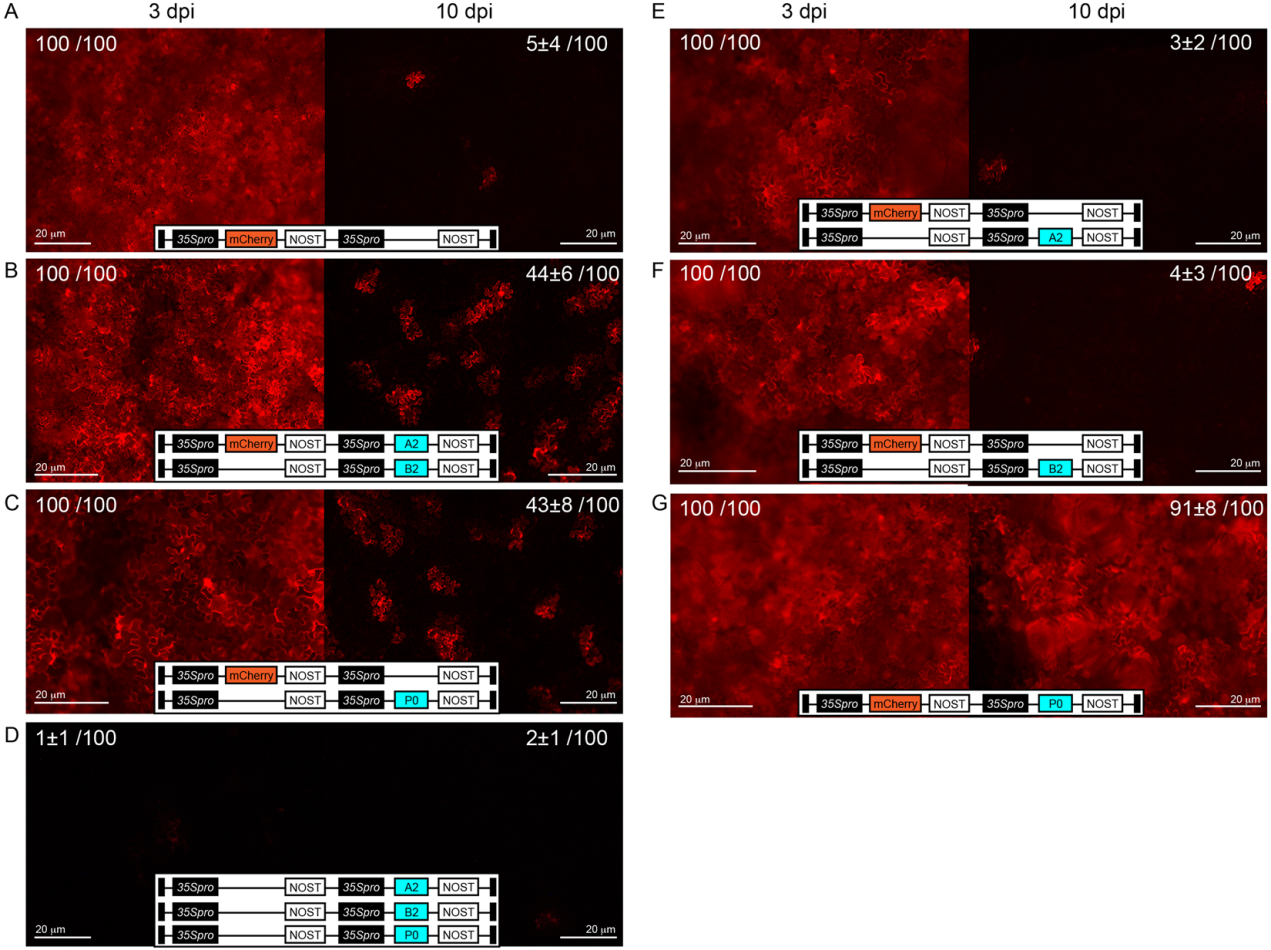
Effect of split PLP0 on post-agroinfiltration analysis. (**A**–**G**) *N. benthamiana* leaves infiltrated with constructs, represented in white boxes, were observed for mCherry fluorescence at 3 and 10 days post-infiltration (dpi). Percentage of cells showing mCherry fluorescence, in three biological replicates, is given for each agroinfiltration. Optical density (OD) was 0.2 per construct. Scale bars, 20 μm.

Silencing suppressors are widely used in co-infiltration assays as powerful tools to enhance and extend the duration of protein accumulation. We engineered two plasmids, each containing one part of the split PLP0, and included an expression cassette for mCherry in one of the plasmids. These plasmids were then used to test whether the split PLP0 system can enhance and extend the duration of mCherry accumulation. We co-infiltrated both constructs and analyzed the mCherry fluorescence at 3 and 10 days post-infiltration (dpi) (Fig. 2). At 3 dpi, co-infiltrations with the vector containing the mCherry cassette showed fluorescence in all infected cells as well as visibly brighter fluorescence in the presence of both parts of PLP0 split as well as the full-length PLP0 (Fig. 2). The increased fluorescence levels were in agreement with that observed for GFP (Fig. 1C). At 10 dpi, the host silencing machinery effectively inhibited mCherry expression in the absence of PLP0. Only cells co-infiltrated with both halves or with the full-length PLP0 construct retained high levels of mCherry, although apparently higher with constructs harboring both mCherry and PLP0 full-length. These results demonstrate that both split PLP0 and full-length gene expression are effective in extending the duration of mCherry accumulation. Importantly, silencing suppression activity was not observed upon co-infiltration with either half of PLP0; i.e., split of PLP0 resulted in two proteins that do not have silencing suppression activity alone (Fig. 2E,F).

### Q system expression in *N. benthamiana* leaves

To test the split PLP0 proteins in a complex set up involving co-expression of multiple genes, we first established a binary expression system for transient expression in *N. benthamiana*. The Q system is a commonly used binary system in insects and vertebrates, but has not been reported in plants. This system utilizes regulatory genes from the *qa* gene cluster of *Neurospora crassa*, which consists of four structural genes (*qa-2, qa-3, qa-4, qa-y*) and two regulatory genes (*qa-1F and qa-1S*) involved in the catabolism of quinic acid^9,10^. QA-1F (hereafter QF) is a transcriptional activator that binds to a 16-base-pair (bp) sequence present upstream of each *qa* gene^11^, while QA-1S (hereafter QS) is a repressor of QF^12^.

**Figure 3:**
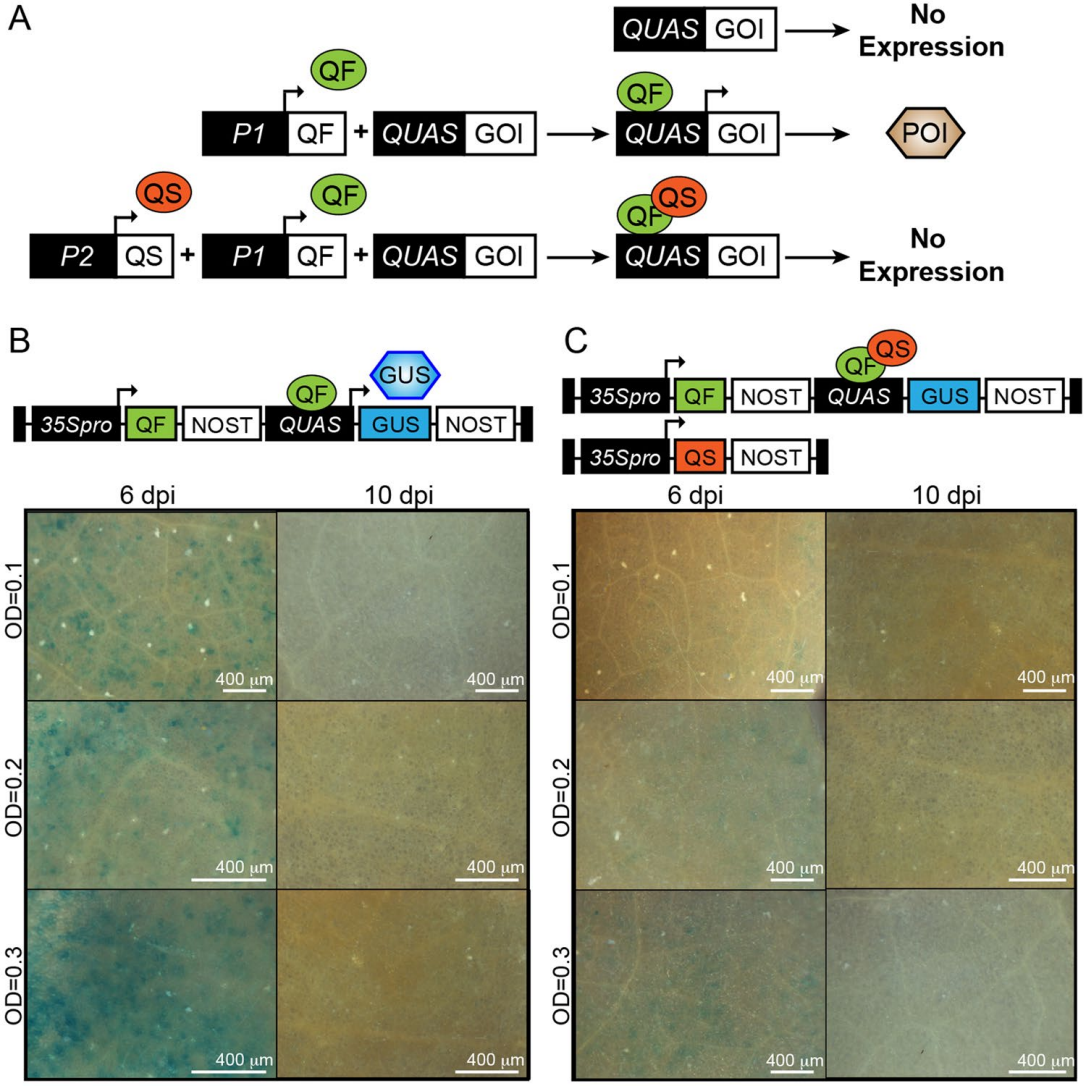
Schematic of Q system components and their expression in *N. benthamiana* leaves. (**A**) Schematic representing the function of the Q system components (modified from Potter & Luo 2011). The QUAS promoter is fused to a gene of interest (GOI) and, in the presence of QF transcription factor, is active, leading to the production of a cognate protein of interest (POI). QS, however, represses QF transcriptional activity; hence, in the presence of QS, QUAS is inactive. P1 and P2 indicate promoter regions. (**B**) QUAS was fused to *β-glucuronidase* (GUS) in a binary vector constitutively expressing QF (top of panel). Agroinfiltrated *N. benthamiana* leaves were stained for GUS activity at the indicated bacterial optical density (OD) per construct at 6 and 10 days post-infiltration (dpi). (**C**) A binary vector constitutively expressing QS was used in combination with QS/QUAS/GUS vector (top of panel). Agroinfiltrated leaves were stained for GUS as indicated. Scale bars, 400 μm.

We used the artificial promoter QUAS, harboring five copies of the QF binding site^13^, to drive gene expression under control of the QF-QS binary system (Fig. 3A). The *β-glucuronidase* (*GUS*) gene driven by the QUAS promoter was cloned in a construct that also contained a *QF* gene driven by the CaMV 35 S promoter. In a separate construct, the *QS* gene was expressed under the control of the 35 S promoter. Agroinfiltration of *N. benthamiana* leaves with the *QF* gene and QUAS promoter-driven *GUS* resulted in GUS-stained cells at 6 dpi, but not at 10 dpi. This suggests that the transcriptional activation component of the Q system (i.e., *QF* gene and QUAS promoter) are active in *N. benthamiana* leaves and, as expected, their expressions are prone to silencing as evidenced by loss of GUS expression in leaves analyzed after 10 dpi (Figs 3B and S2). Upon co-infiltration with *QS*, GUS staining was strongly suppressed at 6 dpi and 10 dpi, suggesting that the repressive component of the Q system (i.e., the *QS* gene) is also active (Fig. 3C). However, this suppression was not complete as evidenced by few stained cells at 6 dpi, particularly at higher ODs, suggesting that some cells expressed the transcriptional activation component of the Q system, but not its repressor. Altogether, these results demonstrate that the use of the strong constitutive 35 S driving the expression of QF and QS is sufficient to recapitulate the Q system in plants. We term this plant-modified Q system: pQ.

### Expression of pQ system coupled with the split silencing suppressor

**Figure 4:**
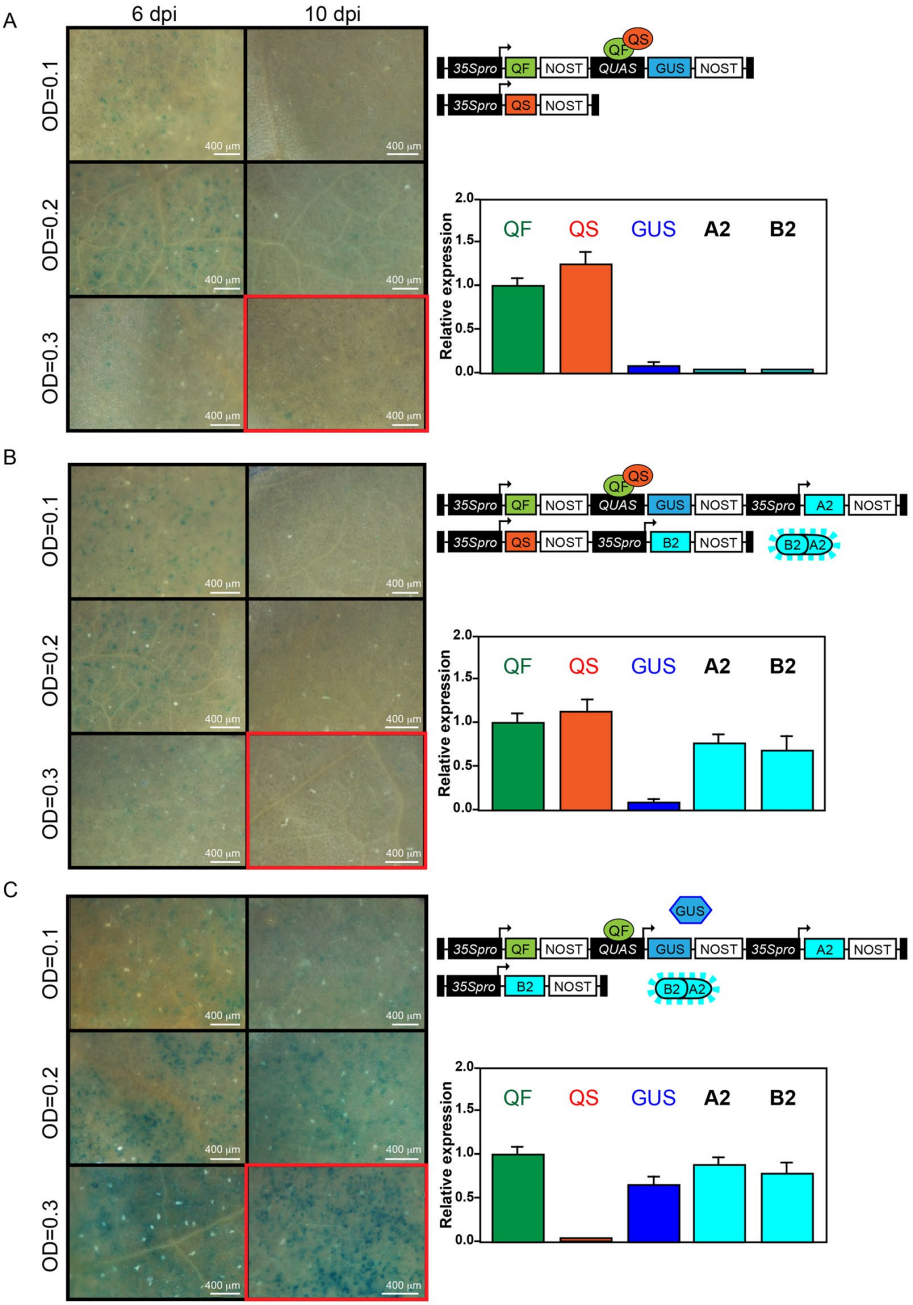
Effect of split PLP0 on the co-infiltration of Q system components. (**A**–**C**) *N. benthamiana* leaves, infiltrated with constructs represented in the upper-right corner of each panel, were stained for GUS activity (left of each panel). Transcript levels were determined for QF, QS GUS, PLP0-A2 (A2) and PLP0-B2 (B2) (bottom-right corner of each panel) for samples infiltrated at optical density (OD) 0.3 at 10 days post-infiltration (dpi) (red boxes). Transcript levels relative to QF expression. Scale bars, 400 μm.

Although the pQ system is active in *N. benthamiana* leaves, its analysis can be biased by partial suppression of QF by QS in co-infiltrations. Since we observed a complete silencing of GUS expression after 10 dpi, we reasoned that coupling the pQ system with the split silencing suppressor PLP0 may result in cells that only co-express the pQ components, rather than a chimeric transfected leaf. Thus, the constructs shown in Fig. 2 were modified to include an expression cassette for QF and QUAS promoter-driven GUS (together with a cassette for PLP0-A2) in one construct, and an expression cassette for QS (together with a cassette for PLP0-B2) in the other construct (Fig. 4). These constructs were used for agroinfiltration of *N. benthamiana* leaves, and GUS staining and transcript levels were analyzed. Similar to the results shown in Fig. 3, in the presence of QS, GUS stained cells were almost completely suppressed by host silencing at 6 dpi and not detected at 10 dpi. Furthermore, at 10 dpi and highest bacterial optical density, the transcript levels of each component (i.e., QF, QS, GUS, A2 and B2) tightly correlated with the GUS staining. That is, in the presence of QS, QF was expressed to high levels but GUS transcripts were not detected, irrespectively of PLP0-A2/B2 expression, whereas in the absence of QS, GUS transcript levels were readily detected and the GUS staining was evident in the presence of PLP0-A2 and PLP0-B2. These results show that co-infiltration analysis of the binary pQ system can be improved when coupled with the split silencing suppressor PLP0-A2/B2; this is because all pQ components appear tightly expressed together in same cells. Altogether, these results demonstrate that the split PLP0 system is active in co-infiltrations involving multiple genes.

## Discussion

Plants have evolved complex and sophisticated mechanisms to fight virus infections, such as RNA silencing through production of siRNAs against the virus genome. About two thirds of plant viruses have RNA genomes and replicate by producing dsRNAs; hence they are obvious substrates for dicer proteins, triggering the host’s silencing machinery. However, plant viruses often thrive in nature because they produce silencing suppressor proteins that inhibit various steps of the host’s silencing pathways.

Poleroviruses encode a potent silencing suppressor, P0, which plays an essential role in viral RNA accumulation even though its poor translation hinders its protein detection in infected plants^14,15^. P0 is an F-box protein that interacts with the host S-phase kinase-associated protein1 (SKP1), forming functional Skp, Cullin, F-box containing (SCF) complexes, to promote ubiquitination of AGO1^5,6,8^. However, P0-induced ubiquitination of AGO1 leads to autophagy and is proteasome-independent^4^. F-box proteins provide ubiquitination target specificity to SCF complexes by interacting with specific partner proteins. Hence, F-box proteins must harbor at least two surfaces for interaction; i.e., one to interact with Skp and another with their targets. Here we showed that, consistent with an F-box having two interaction surfaces, PLP0 can be split into two proteins that are only functional as silencing suppressors upon co-expression. This suggests that both split parts of PLP0 are independently folded into their native structures, and that each folded part interacts with the other to form a functional PLP0 protein.

**Figure 5:**
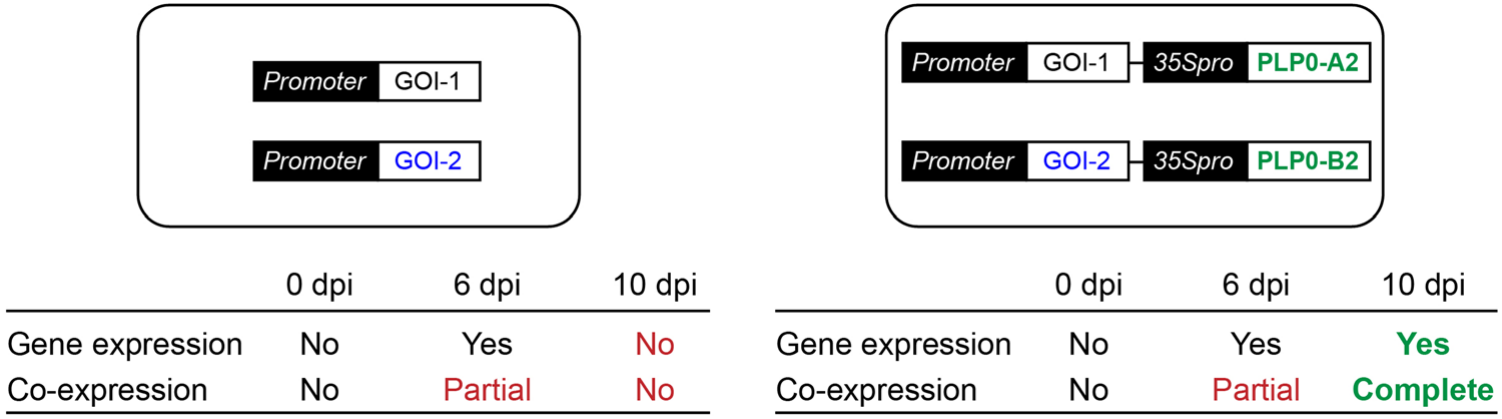
Schematic of the conditional silencing suppression system, split PLP0. In the absence of the split PLP0 system (left panel), co-infiltration of two genes of interest (GOI-1 and GOI-2) results in expression of both genes at 6 days post-infiltration (dpi); however, their co-expression is only partial (i.e., a number of cells only express GOI-1 or GOI-2). Complete co-expression is obtained using the conditional silencing suppression system (right panel). Constructs expressing GOI-1 and GOI-2 are modified to also express PLP0-A2 and PLP0-B2, respectively, and are analyzed at 10 dpi, resulting in analysis of only cells that express both genes of interest.

We also demonstrated that the split PLP0 can be used as a conditional silencing suppression system in transient expression using *N. benthamiana* leaves (Fig. 5). After silencing occurs (in the absence of silencing suppressor) in agroinfiltrated leaves, the use of this conditional suppression system appears to restrict gene expression to only cells that express both parts of PLP0. This property of the PLP0 conditional silencing suppression may be useful in future research requiring tight transient co-expression.

In addition to a conditional silencing suppression system, we showed that the pQ system is active in transient assays, using the 35 S promoter to drive QF and QS. This system will likely operate in stable transgenic lines utilizing either the 35 S or other promoters. There are few transcriptional repressor systems available for plant molecular biology. A system incorporating the *Tet* repressor and a modified 35 S promoter was developed many years ago^16^ and demonstrated to work in tobacco and potato but it has been reported to be toxic in Arabidopsis and tomato^17^. With this limitation, chemical-induced promoters (e.g., dexamethasone or alcohol) have been favoured in recent times^18^. If the pQ-system is functional and benign in a wide range of plant species, it has the potential to play an enabling role in molecular plant biology where rapid, controllable and tight switching off of gene expression is required.

## Materials and Methods

### Plant material and experimental conditions

*N. benthamiana* plants were grown in standard greenhouse conditions (23–25 °C, cycle of 16 h of light and 8 h of darkness) for approximately 4 weeks before the agroinfiltration assays.

### Plasmids

Plasmids were produced using pORE-O1, a multi-cloning site vector^19^. The CaMV 35 S promoter was amplified from pORE-O1 and used to replace the hydroperoxide lyase promoter via *Aat*II and *Fse*I restriction digestion followed by ligation. PLP0 full-length and truncations were cloned via *Fse*I and *Asc*I restriction digestion followed by ligation. mCherry, QF-QUAS-GUS and QS were cloned using *Nhe*I and *Eco*RI. Primers are listed in Table S2.

### Transient Expression Assays

*Agrobacterium* infiltration (agroinfiltration) was performed as previously described^20^. Approximately 4-week-old *N. benthamiana* leaves were agroinfiltrated using equal volumes of *Agrobacterium* cultures, each containing the desired binary plasmid mixed prior to co-infiltration. Final dilutions of cultures used in co-infiltration assays were as stated in each figure and agroinfiltrations were performed in triplicate.

### RNA extraction and quantitative PCR (qPCR)

Total RNA was extracted using TRIzol Reagent (Life Technologies) according to the manufacturer’s instructions. For qPCR, cDNA was produced from 1 μg of DNase-treated total RNA using Superscript III reverse transcriptase (Life Technologies) according to the manufacturer’s protocol. qPCR was performed using Brilliant II SYBR Green QPCR Master Mix (Agilent Technologies) in an Mx3000P instrument (Agilent Technologies), following the manufacturers’ instructions. Each qPCR reaction was performed in both biological and technical triplicate using *Cyclophilin 5* (AT2G29960) as a gene expression normalizer. Data was analyzed with MxPro QPCR Software (Agilent Technologies).

### Protein structure prediction

Secondary structure prediction for PLP0 protein was performed using the I-TASSER online server with default parameters^21^.

### Data availability

Plasmids will be provided upon request.

## Electronic supplementary material

Dataset 1
