# Supplementary material for: A conditional silencing suppression system for transient expression
Source: Sci Rep. 2018 Jun 21;8:9426. doi: 10.1038/s41598-018-27778-3 (PMC6013485; doi:10.1038/s41598-018-27778-3)
Supplement: Supplementary file 1 — Dataset 1 [file 41598_2018_27778_MOESM1_ESM.docx]

**SUPPLEMENTARY INFORMATION FILES**

**A conditional silencing suppression system for transient expression**

Rodrigo Siqueira Reis^1,2,*^, Celso G. Litholdo Jr.^2,3^, Julia Bally^2,5^, Thomas H. Roberts^4^, Peter M. Waterhouse^2,5^

^1^ Department of Plant Molecular Biology, University of Lausanne, Lausanne 1015, Switzerland

^2^ School of Life and Environmental Sciences, University of Sydney, NSW 2006, Australia

^3^ Citrus Biotechnology Lab, Centro de Citricultura, Instituto Agronômico de Campinas, Cordeirópolis SP 13490-000, Brazil

^4^ Plant Breeding Institute, Sydney Institute of Agriculture, University of Sydney, NSW 2006, Australia

^5^ Centre for Tropical Crops and Biocommodities, Queensland University of Technology, Brisbane QLD 4001, Australia

* Corresponding author (Email: [Rodrigo.SiqueiraReis@unil.ch](mailto:Rodrigo.SiqueiraReis@unil.ch); Twitter: @Rodrigo_S_Reis)


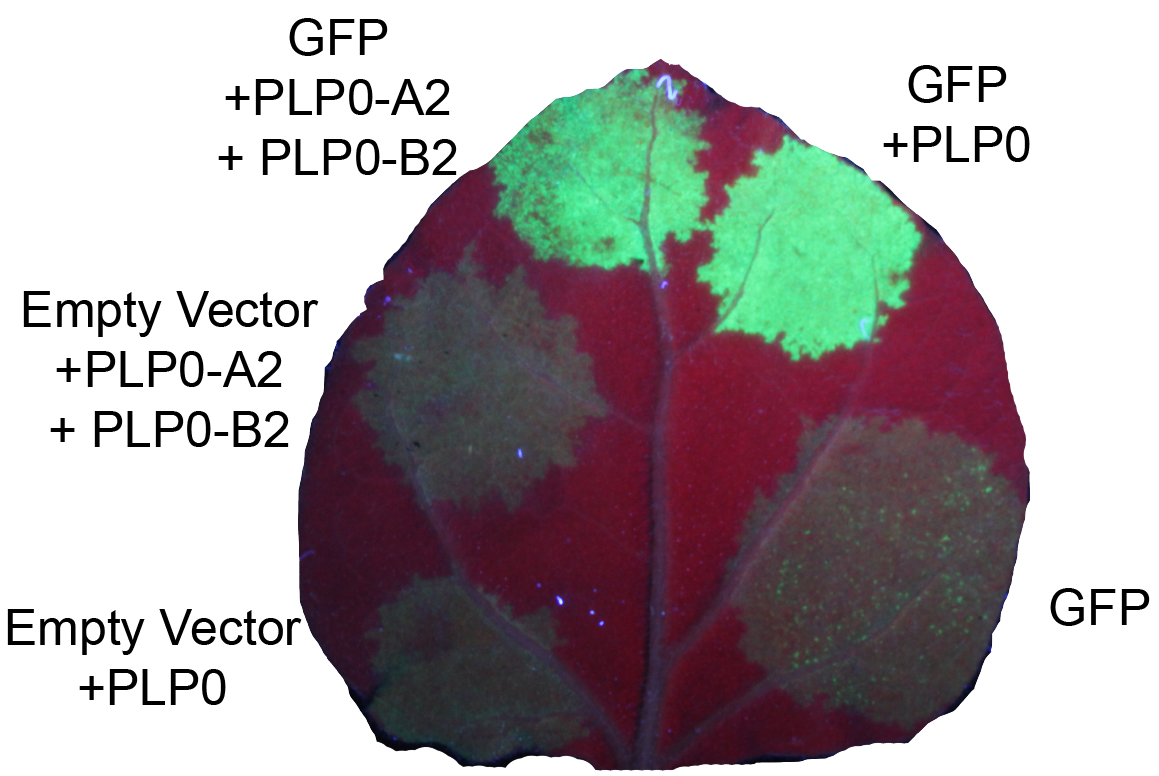


**Fig. S1**. Co-infiltration of GFP with PLP0 and each combination of PLP0-A and B in *N. benthamiana* leaves agroinfiltrated at an OD of 0.2 per construct and analysed at 5 dpi.

**
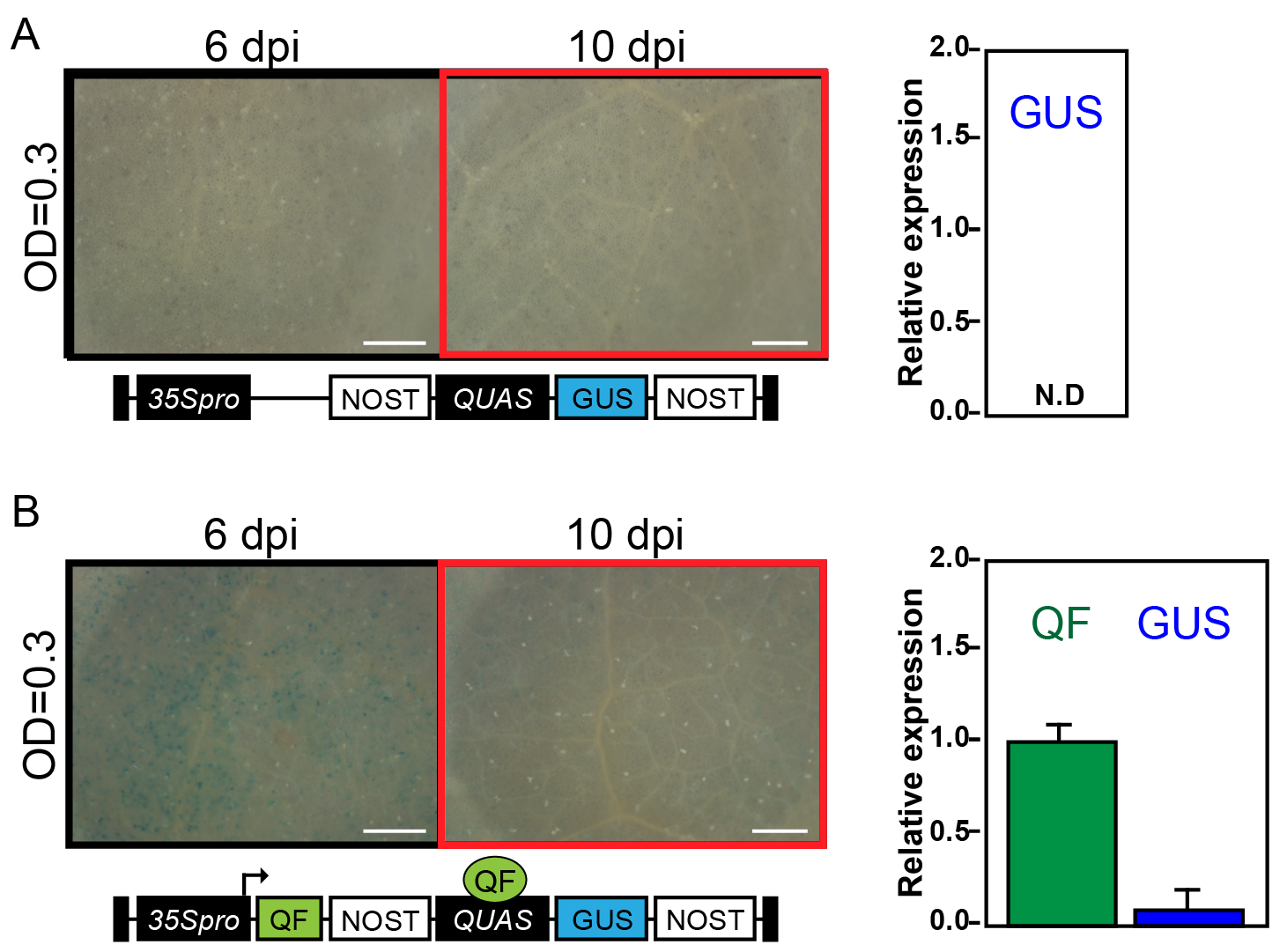
**

**Fig. S2**. Presence of Q system components and GUS expression. (A) GUS staining and expression in the absence of QS and QF. (B) GUS staining and expression in the presence of QF and absence of QS. Scale bars, 400 μm. Transcript levels are shown for each co-infiltration at bacterial OD 0.3 and 10 days dpi (highlighted in a red square; left panel). N.D, not detected.

**Table S1**. List of PLP0 amino acid sequences used for alignments.

>gi|18656710|gb|AAL77921.1|AF453389_1 P0 [Potato leafroll virus]

MIVLTQSGTLLFDQRFKLSKFLFVVIATGFPLLLQQASLIYGYNHEQIYR

ICRSFLYVLPLLNCKRGRISTSGLQLPRHLHYECLEWGLLCGTHPAIQIV

GPTIVIKLDDPTTAAAYRSELLRVSSSSYIQNAAGLSNGWGHDMEAFVRN

AICLLELRERSIPQSGLRDLMGNYQHLVRSLLDACEVDHFVPLDFQHRSL

MLNFARLYNQLDLQGRAKSFRALTGFPVYVPSEDYLEGSFLQKELQE

>gi|55275985|gb|AAV49758.1| P0 [Potato leafroll virus]

MIVLTQSGTLLFDQRFKLSKFLFVVIATGFPLLLQQASLIYGYNHEQIYR

ICRSFLYVLPLLNCKRGRISTSGLQLPRHLHYECLEWGLLCGTHPAIQIV

GPTIVIKLDDPTTAAAYRSELLRVSSSSYIQNAAGLSNGWGHDMEAFVRN

AICLLELRERSIPQSGLRDLMGNYQHLVRSLLDACEVDHFVPLDFQHRSL

MFNFARLYNQLDLQGRAKSFRALTGFPVYVPSEDYLEGSFLQKELQE

>gi|517276|emb|CAA52789.1| unnamed protein product [Potato leafroll virus]

MIVLTQSGTLLFDQRFKLSKFLFVVIATGFPLLLQQASLIYGYNHEQIYR

ICRSFLYVLPLLNCKRGRISTSGLQLPRHLHYECLEWGLLCGTHPAIQIV

GPTIVIKLDDPTTAAAYRSELLRVSSSSYIQNAAGLSNGWGHDMEAFVRN

AICLLELRERSIPQSGLRDLMGNYQHLVRSLLDACKVDHFVPLDFQHRSL

MLNFARLYNQLDLQGRAKSFRALTGFPVYVPSEDYLEGSFLQKELQE

>gi|18479113|gb|AAL73421.1|AF453401_1 P0 [Potato leafroll virus]

MIVLTQSGTLLFDQRFKLSKFLFVVIATGFPLLLQQASLIYGYNHEQIYR

ICRSFLYVLPLLNCKRGRISVSGLQLPRHLHYECLEWGLLCGTHPAIQIV

GPTIVIKLDDPTTAAAYRSELLRVSSSSYIQNAAGLSNGWGYDMEAFVRN

AICLLELRERSIPQSGLRDLMGNYQHLVRSLLDACKVDHFVPLDFQHRSL

MLNFARLYNQLDLQGRAKSFRALTGFPVYVPSEDYLEGSFLQKELQE

>gi|18656701|gb|AAL77913.1|AF453388_1 P0 [Potato leafroll virus]

MIVLTQSGTLLFDQRFKLSKFLFVVIATGFPLLLQQASLIYGYNHEQIYR

ICRSFLYVLPLLNRKRGRISTSGLQLPRHLHYECLEWGLLCGTHPAIQIV

GPTIVIKLDDPTTAAAYRSELLRVSSSSYIQNAAGLSNGWGHDMEAFVRN

AICLLELRERSIPQSGLRDLMGNYQHLVRSLLDACKVDHFVPLDFQHRSL

MLNFARLYNQLDLQGRAKSFRALTGFPVYVPSEDYLEGSFLQKELQE

>gi|18479129|gb|AAL73431.1|AF453406_1 P0 [Potato leafroll virus]

MIVLTQSGTLLFDQRFKLSKFLFVVIATGFPLLLQQASLIYGYNHEQIYR

ICRSFLYVLPLLNCKRGRISTSGLQLPRHLHYECLEWGLLCGTHPAIQIV

GPTIVIKLDDPTTAAAYRSELLRVSSSSYIQNAAGLSNGWGHDMEAFVRN

AICLLELRERSIPQSGLRDLMGNYQHLVRSLLDACEVDHFVPLDFQHRSL

MLNFARLYNQLDLQGRAKSFRALTGFPIYVPSEDYLEGSFLQKELQE

>gi|55275969|gb|AAV49750.1| P0 [Potato leafroll virus]

MIVLTQSGTLLFDQRFKLSKFLFVVIATGFPLLLQQASLIYGYNHEQIYR

ICRSFLYVLPLLNCKRGRISTSGLQLPRHLHYECLEWGLLCGTHPAIQIV

GPTIVIKLDDPTTAATYRSELLRVSSSSYIQNAAGLSNGWGHDMEAFVRN

AICLLELRERSIPQSGLRDLMGNYQHLVRSLLDACEVDHFVPLDFQHRSL

MLNFARLYNQLDLQGRAKSFRALTGFPVYVPSEDYLEGSFLQKELQE

>gi|9629161|ref|NP_056746.1| P0 protein [Potato leafroll virus]

MIVLTQSGTLLFDQRFKLSKFLFVVIATGFPLLLQQASLIYGYNHEQIYR

ICRSFLHVLPLLNCKRGRISTSGLQLPRHLHYECLEWGLLCGTHPAIQIV

GLTIVIKLDDPTTAAAYRSELLRVSSSSYIQNAAGLSNGWGHDMEAFVRN

AICLLELRERSIPQSGLRDLMGNYQHLVRSLLDACKVDHFVPLDFQHRSL

MLNFARLYNQLDLQGRAKSFRALTGFPVYVPSEDYLEGSFLQKELQE

>gi|137267|sp|P11621.1|P0_PLRVW RecName: Full=Suppressor of silencing P0; AltName: Full=28 kDa protein; AltName: Full=Protein ORF0

MIVLTQSGTLLFDQRFKLSKFLFVVIATGFPLLLQQASLIYGYNHEQIYR

ICRSFLYILPLLNCKRGRISTSGLQLPRHLHYECLEWGLLCGTHPAIQIV

GPTIVIKLDDPTTAAAYRSELLRVSSSSYIQNAAGLSNGWGHDMEAFVRN

AICLLELRERSIPQSGLRDLMGNHQHLVRSLLDACKVDHFVPLDFQHRSL

MLNFARLYNQLDLQGRAKSFRALTGFPVYVPSEDYLEGSFLQKELQE

>gi|185534897|gb|ACC77817.1| P0 protein [Potato leafroll virus]

MIVLTQSGTLLFDQRFKLSKFLFVVIATGFPLLLQQASLIYGYNHEQIYR

ICRSLLYILPLLNCKRGRISTSGLQLPRHLHYECLEWGLLCGTHPAIQIV

GPTIVIKLDDPTTAAAYRSELLRVSSSSYIQNAAGLSNGWGHDMEAFVRN

AICLLELRERSIPQSGLRDLMGNHQHLVRSLLDACKVDHFVPLDFQHRSL

MLNFARLYNQLDLQGRAKSFRALTGFPVYVPSEDYLEGSFLQKELQE

>gi|18479110|gb|AAL73419.1|AF453400_1 P0 [Potato leafroll virus]

MIVLTQSGTLLFDQRFKLSKFLFVVIATGFPLLLQQASLIYGYNHEQIYR

ICRSFLYVLPLLNCKRGRISTSGLQLPRHLHYECLEWGLLCGTHPAIQIV

GPTIVIKLDDPTTAAAYRSELLRISSSSYIQNAVGLSNGWGHDMEAFVRN

AICLLELRERSIPQSGLRDLMGNYQHLVRSLLDACKVDHFVPLDFQHRSL

MLNFARLYNQLDLQGRAKSFRALTDFPVYVPSEDYLEGSFLQKELQE

>gi|18656754|gb|AAL77960.1|AF453394_1 P0 [Potato leafroll virus]

MIVLTQSGTLLFDQRFKLSKFLFVVIATGFPLLLQQASLIYGYNHEQIYR

ICRSFLYVLPLLNCKRGRISTSGLQLPRHLHYECLEWGLLCGTHPAIQIV

GPTIVIKLDDPTTAAAYRSELLRVSSSSYIQNAAGLSNGWGHDMEAFVRN

AICLLELRERSIPQSGLRDLMGNYQHLVRSLLDACKVDHFVPLDFQHRSL

MLNLARLYNQLDLQGRAKSFRALTGFPVYVPSEDYLEGSSLQKELQEREG

CGRIQRVFGPTETAKVCRN

>gi|18479116|gb|AAL73423.1|AF453402_1 P0 [Potato leafroll virus]

MIVLTQSGTLLFDQRFKLSKFLFVVIATGFPLLLQQASLIYGYNHEQIYR

ICRSFLYVLPLLNCKRGRISTSGLQLPRHLYYECLEWGLLCGTHPAIQIV

GPNIVIKLDDPTTAAAYRSELLRVSSSSYIQNAAGLSNGWGHDMEAFVRN

AICLLELRERSIPQSGLRDLMGNYQHLVRSLLDACKVDHFVPLDFQHRSL

MLNFARLYNQLDLQGRAKSFRALTGLPVYVPSEDYLEGSFLQKELQE

>gi|18656719|gb|AAL77929.1|AF453390_1 P0 [Potato leafroll virus]

MIVLTQSGTLLFDQRFKLSKFLFVVIATGFPLLLQQASLIYGYNHEQIYR

ICRSLLYVLPLLNCKGGRISTSGLQLPRHLHYECLEWGLLCGTHPAIQIV

GSTIVIKLDDPTTAAAYRSELLRVSSSSYIQNAAGLSNGWGHDMEAFVRN

AICLLELRERSIPQSGLRDLMGNYQHLVRSLLDACEVDHFVPLDFQHRSL

MLNFARLYNQLDLQGRAKSFRALTGFPVYVPSEDYLEGSFLQKELQE

>gi|188011178|gb|ABY49845.2| P0 [Potato leafroll virus]

MIVLTQSGTLHFDQRFKLSKFLFVVIATGFPLLLQQASLIYGYNHEQIYR

ICRSFLYVLPLLNCKRGRISTSGLQLPRHLHYECLEWGLLCGTHPAIQIV

GPTIVIKLDDPITAAAYRSELLRVSSSSYIQNAAGLSNGWGHDMEAFVRN

AICLLELRERSIPQSGLRDLMGNYQHLVRSILDACEVDHFVPLDFQHRSL

MLNFARLYNQLDLQGRAKSFRALTGFPVYVPSEDYLEGSFLQKELQE

>gi|18479094|gb|AAL73409.1|AF453395_1 P0 [Potato leafroll virus]

MIVLTQSGTLLFDQRFKLSKFLFVVIATGFPLLLQQASLIYGYSHEQIYR

ICRSFLYVLPLLNCKRGRISTSGLQLPRHLHYECLEWGLLCGTHPAIQIV

GPTIVIKLDDPTTAAAYRSELLRVSSSSYIQNAAGLSNGWGHDMEAFVRN

AICLLELRERSIPQSGLRNLMGNYQHLVRSLLDACQVDHFVPLDFQHRSL

LLNFARLYNQLDLQGRAKSFRALTGFPVYVPSEDYLEGSFLQKELQE

>gi|18656745|gb|AAL77952.1|AF453393_1 P0 [Potato leafroll virus]

MIVLTQSGTLLFDQRFKLSKFLFVVIATGFPLLLQQASLIYGYNHEQIYR

ICRSFLYVLPLLNCKRGRISTSGLQLPRHLHYECLEWGLLCGTHPAIQIV

GPTIVIKLDDPTTAAAYRSELLRVSSSSYIQNAAGLSNGWGHDMEAFVRN

AICLLELLERNIPQSGLRDLMGNHQHLVRSLLDACEVDHFVPLDFQHRSL

MLNFARLYNQLDLQGRAKSFRALTGFPVYVPSEDYLEGSLLQKELQE

>gi|18479103|gb|AAL73415.1|AF453398_1 P0 [Potato leafroll virus]

MIVLTQSGTLLFDQRFKLSKFLFVVIATGFPLLLQQASLIYGYSHEQLHR

ICRSFLYVLPLLNCKRGRISTAGLQLPRHLHYECLEWGLLCGTHPAIQIV

GPTIVIKLDDPTTAAAYRSELLRVSSSSYIQNAAGLSNGWGHDMEAFVRK

AICLLELRERSIPQSGLRNLMGNYQHLVRSLLDACQVDHFVPLDFQHRSL

LLNFARLYNQLDLQGRAKSFRALTGFPVYVPSEDYLEGSFLQKELQE

>gi|18479106|gb|AAL73417.1|AF453399_1 P0 [Potato leafroll virus]

MIVLTQSGTLLFDQRFKLSKFLFVVIATGFPLLLQQASLIYGYNHEQIYR

ICRSFLYVLPLLNCKRGRISTSGLQLPRHLHYECLEWGLLCGTHPAIQIV

GPTIVIKLDDPTTTAAYRSELLRVSSSSYIQNAAGLSNGWGHDMETFVRN

AICLLELRERSIPQSSLRDLMGNYQHLVRSLLDACEVDHSVPLDFQHRSL

MLNLARLYNQLDLQGRAKSFRALTGFPVYVPSEDYLEGSFLQKELQE

>gi|18479100|gb|AAL73413.1|AF453397_1 P0 [Potato leafroll virus]

MIVLTQSGTLLFDQRFKLSKFLFVVIATGFPLLLQQASSIYGYNHEQIYR

ICRSFLYVLPLLNCKRGRISTSGLQLPRHLHYECLEWGLLCGTHPAIQIV

GPTIVIKLDDPTTAAAYRSELLRISSSSYIQNAAGLSNGWGHDMEAFVRN

AICLLELRERSIPQSGLRNLMGNYQHLVRSLLDACQVDHFVPLDFQHRSL

LLNFARLCNQLDLQGRARSFRALTGIPIYVPYEDYLEGSFLQKELQE

>gi|18479097|gb|AAL73411.1|AF453396_1 P0 [Potato leafroll virus]

MIVLTQSGILLFDQRFKLSKFLFVVIATGFPLLLQQASLIYGYSHEQHHR

ICRSFLYVLPLLNCKRGRISTSGLQLPRHLHYECLEWGLLCGTHPAIQIV

GPTIVIKLDDPTTAAAYRSELLRVSSSSYIQNAAGLSNGWGHDMEALVRN

AICLLELRERSIPQSGLRNLMGNYQHLVRSLLDACQVDHFVPLDFQHRSL

LLNFARLYNQLDLQGRAKSFRALTGIPIYVPSEDYLEGSFLQKELQE

>gi|55275977|gb|AAV49754.1| P0 [Potato leafroll virus]

MIVLTQSGTLLFDQRFKLSKFLFVVIATGFPLLLQQASLLYGYNHEQIYR

ICRSFLYVLPLLNCKRGRISTSGLQLPRHLHYECLEWGLLCGTHPAIQIV

GPSIIVELDNPTTAAAYRSELLRISSSSYVKNSVGFSNGWGHDMEAFVRN

AICLLELRERSIPQSGLRNLMGNYQHLVRSLLDACTVDYFVPLDFQHRSL

MLNFARLYNQLDLQGRAKSFRALTGFPVYVPSEDYLEGSFLQKELQE

>gi|55275975|gb|AAV49753.1| P0 [Potato leafroll virus]

MIVLTQSGTLLFDQRFKLSKFLFVVIATGFPLLLQQASLLYGYNHEQIYR

ICRSFLYVLPLLNCKRGRISTSGLQLPRHLHYECLEWGLLCGTHPAIQIV

GPSIIVELDNPTTAAAYRSELLRISSSSYVKNSVGYSNGWGHDMEAFVRN

AICLLELRERSIPQSGLRNLMGNYQHLVRSLLDACTVDYFVPLDFQHRSL

MLNFARLYNQLDLQGRAKSFRALTGFPVYVPSEDYLEGSFLQKELQE

>gi|18656737|gb|AAL77945.1|AF453392_1 P0 [Potato leafroll virus]

MIVLTQSGTLLFDQRFKLSKFLFVVIATGFPLLLQQASLLYGYNHEQIYR

ICRSFLYVLPLLNCKRGRISTSGLQLPRHLHYECLEWGLLCGTHPAIQIV

GPSIIVELDNPTTAAAYRSELLRISSSSYVKNSVGSSNGWGHDMEAFVRN

AICLLELRERSIPQSGLRNLMGNYQHLVRSLLDACTVDYFVPLDFQHRSL

MLNFARLYNQLDLQGRAKSFRALTGFPVYVPSEDYLEGSFLQKELQE

>gi|55275971|gb|AAV49751.1| P0 [Potato leafroll virus]

MIVLTQSGTLLFDQRFKLSKFLFVVIATGFPLLLQQASLLYGYNHEQIYR

ICRSFLYVLPLLNCKRGRISTSGLQLPRHLHYECLEWGLLCGTHPAIQIV

GPSIIVELDNPTTAAAYRSELLRISSSSYVKNSVGFSNGWGHDMEAFVRN

AICLLELRERSIPQSGLRNLMGNYQHLVRSLLDACTVDYFVPLDFQHRSL

MLNFARLYNQLDLQGRAKSFRALTGFPVYVPSEDYLEDSFLQKELQE

>gi|55275983|gb|AAV49757.1| P0 [Potato leafroll virus]

MIVLTQSGTLLFDQRFKLSKFLFVVIATGFPLLLQQASLLYGYNHEQIYR

ICRSFLYVLPLLNCKRGRISTSGLQLPRHLHYECLEWGLLCGTHPAIQIV

GPSIIVELDNPTTAAAYRSELLRISSSSYVKNSVGFSNGWGHDMEAFVRN

AICLLELRERSIPQSGLRNLMGNYQHLVRSLLDACTVDHFVPLDFQHRSL

MLNFARLYNQLDLQGRAKSFRALTGFPVYVPSEDYLEGSFLQKELQE

>gi|55275981|gb|AAV49756.1| P0 [Potato leafroll virus]

MIVLTQSGTLLFDQRFKLSKFLFVVIATGFPLLLQQASLLYGYNHEQIYR

ICRSFLYVLPLLNCKRGRISISGLQLPRHLHYECLEWGLLCGTHPAIQIV

GPSIIVELDNPTTAAAYRSELLRISSSSYVKNSVGFSNGWGHDMEAFVRN

AICLLELRERSIPQSGLRNLMGNYQHLVRSLLDACTVDYFVPLDFQHRSL

MLNFARLYNQLDLQGRAKSFRALTGFPVYVPSEDYLEGSFLQKELQE

>gi|18479119|gb|AAL73425.1|AF453403_1 P0 [Potato leafroll virus]

MIVLTQSGTLLFDQRFKLSKFLFVVIATGFPLLLQQASLLYGYNHEQIYR

ICRSFLYVLPLLNCKRGRISTSGLQLPRHLHYECLEWGLLCGTHPAIQIV

GPSIIVELDNPTTAAAYRSELLRISSSSYVKNSVGFSNGWGHDMEAFVRN

AICLLELRERSIPQSGLRNLMGNYQHLVRSLLDACTVDYFIPLDFQHRSL

MLNFARLYNQLDLQGRAKSFRALTGLPVYVPSEDYLEGSFLQKELQE

>gi|18479123|gb|AAL73427.1|AF453404_1 P0 [Potato leafroll virus]

MIVLTQSGTLLFDQRFKLSKFLFVVIATGFPLLLQQASLLYGYNHEQIYR

ICRSFLYVLPLLNRKRGRISTSGLQLPRHLHYECLEWGLLCGTHPAIQIV

GPSIIVELDNLTTAAAYRSELLRISSSSYVKNSVGFSNGWGHDMEAFVRN

AICLLELRERSIPQSGLRNLMGNYQHLVRSLLDACTVDYFIPLDFQHRSL

MLNFARLYNQLDLQGRAKSFRALTGFPVYVPSEDYLEGSFLQKELQE

>gi|18479126|gb|AAL73429.1|AF453405_1 P0 [Potato leafroll virus]

MIVLTQSGTLLFDQRFKLSKFLFVVIATGFPLLLQQASLLYGYNHEQIYR

ICRSFLYVLPLLNCKRGRISTSGLQLPRHLHYECLEWGLLCGTHPAIQIV

GPSIIVELDNPTTAAAYRSELLRISSSSYVKNSAGFSNGWGHDMEAFVRN

AICLLELRERSIPQSGLRNLMGNYNHLVRSLLDACTVDYFVPLDFQHRSL

MLNFARLYNQLDLQGRAKSFRALTGLPVYVPSEDYLEGSFLQKELQE

>gi|55275973|gb|AAV49752.1| P0 [Potato leafroll virus]

MIVLTQSGTLLFDQRFKLSKFLFVVIATGFPLLLQQASSLYGYNHEQIYR

ICRSFLYVLPLLNCKRGRISTSGLQLPRHLHYECLEWGLLCGTHPAIQIV

GPSIIVELDNPTTAAVYRSELLQISSSSYVKNSVGFSNGWGHDMEAFVRN

AICLLELRERSIPQSGLRNLMGNYQHLVRSLLDACTVDYFVPLDFQHRSL

MLNFARLYNQLDLQGRAKSFRALTGFPVYVPSEDYLEGSFLQKELQE

**Table S2**. List of primers.

| Oligo_ID | Oligo_Seq | Purpose |
| --- | --- | --- |
| 35Sp-AatII-F | tttt GACGTC CTCGACGAATTAATTCCAATC | Replace HPLpro with 35Spro |
| 35S-FseI-R | tttt GGCCGGCC CGAGCGTGTCCTCTCCAAATG | Replace HPLpro with 35Spro |
| PLP0-FseI-F | tttt GGCCGGCC ATGATTGTATTGACCCAGTCT | Clone PLP0 or truncation |
| A1-AscI-R | tttt GGCGCGCC TCA GCCCACGATTTGTATAGCG | Clone PLP0 or truncation |
| A2-AscI-R | tttt GGCGCGCC TCA AGGGCCCACGATTTGTATAG | Clone PLP0 or truncation |
| A3-AscI-R | tttt GGCGCGCC TCA GGTAGGGCCCACGATTTGT | Clone PLP0 or truncation |
| A4-AscI-R | tttt GGCGCGCC TCA GTCAAGTTTAATGACGATGG | Clone PLP0 or truncation |
| B1-FseI-F | tttt GGCCGGCC ATG CCTACCATCGTCATTAAACTTG | Clone PLP0 or truncation |
| B2-FseI-F | tttt GGCCGGCC ATG ACCATCGTCATTAAACTTGAC | Clone PLP0 or truncation |
| B3-FseI-F | tttt GGCCGGCC ATG ATCGTCATTAAACTTGACGAC | Clone PLP0 or truncation |
| B4-FseI-F | tttt GGCCGGCC ATG GACCCAACCACTGCCGCC | Clone PLP0 or truncation |
| PLP0-AscI-R | tttt GGCGCGCC TCATTCTTGTAATTCCTTTTGG | Clone PLP0 or truncation |
| Che-NheI-F | tttt GCTAGC ATGGTGAGCAAGGGCGAGGA | Clone mCherry |
| Che-EcoRI-R | tttt GAATTC TTACTTGTACAGCTCGTCCAT | Clone mCherry |
| QF-NheI-F | tttt GCTAGC ATGCCGCCTAAACGCAAGAC | Clone QF-QUAS-GUS cassette |
| GUS-EcoRI-R | tttt GAATTC TCATTGTTTGCCTCCCTG | Clone QF-QUAS-GUS cassette |
| QS-NheI-F | tttt GCTAGC ATGAACACCATCCCGGCAC | Clone QS |
| QS-EcoRI-R | tttt GAATTC TCAAGATATTTGCGTTGCAA | Clone QS |
| P0-HIS-FseI-F | tttt GGCCGGCC TCAGTGGTGATGGTGATGATG ATGATTGTATTGACCCAGTCT | Clone His-tagged PLP0 or truncation |
| P0-HIS-AscI-R | tttt GGCGCGCC TCAGTGGTGATGGTGATGATG TCATTCTTGTAATTCCTTTTGG | Clone His-tagged PLP0 or truncation |
| A2-qPCR-F | TGGAACCTTGCTTTTTGACC | Quantitative PCR (qPCR) |
| A2-qPCR-R | TGAAATCCTGCCTCTTTTGC | Quantitative PCR (qPCR) |
| B2-qPCR-F | CAGCATTTGGTTCGGTCTTT | Quantitative PCR (qPCR) |
| B2-qPCR-R | GAAAGCTGCCCTCCAAATAA | Quantitative PCR (qPCR) |
| QF-qPCR-F | ACACCATGTGAGGGCTTAGG | Quantitative PCR (qPCR) |
| QF-qPCR-R | ACAAAGACGCTGAAGGGAGA | Quantitative PCR (qPCR) |
| QS-qPCR-F | GCTGTCTATGCCATGCTTCA | Quantitative PCR (qPCR) |
| QS-qPCR-R | GTCGGTATGCAGGAAACGAT | Quantitative PCR (qPCR) |
| GUS-qPCR-F | CTGATAGCGCGTGACAAAAA | Quantitative PCR (qPCR) |
| GUS-qPCR-R | GGCACAGCACATCAAAGAGA | Quantitative PCR (qPCR) |
